# Four-Body Nonadditive Potential Energy Surface and the Fourth Virial Coefficient of Helium

**DOI:** 10.1021/acs.jced.3c00578

**Published:** 2023-11-29

**Authors:** Richard J. Wheatley, Giovanni Garberoglio, Allan H. Harvey

**Affiliations:** † School of Chemistry, University of Nottingham, Nottingham NG7 2RD, U.K.; ‡ European Centre for Theoretical Studies in Nuclear Physics and Related Areas (ECT*), Fondazione Bruno Kessler, Trento I 38122, Italy; § Applied Chemicals and Materials Division, National Institute of Standards and Technology, Boulder, Colorado 80305-3337, United States

## Abstract

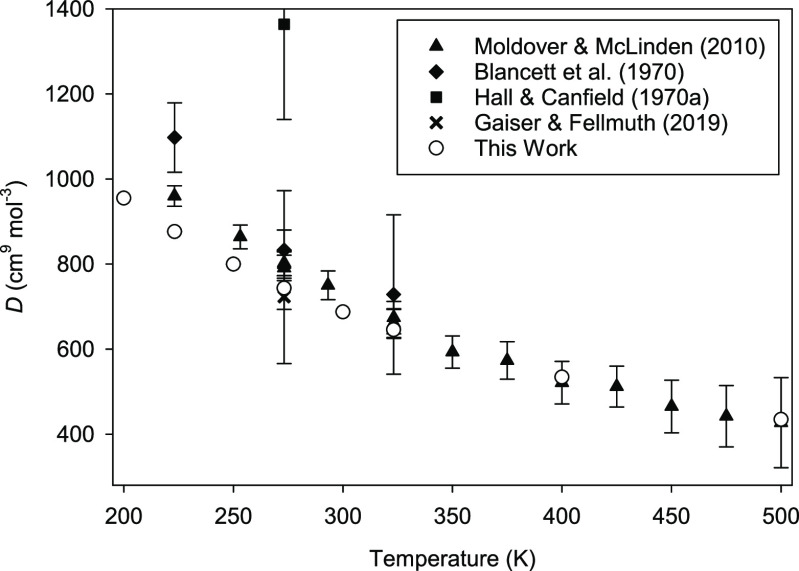

The four-body nonadditive contribution to the energy of four helium atoms is calculated and fitted for all geometries for which the internuclear distances exceed a small minimum value. The interpolation uses an active learning approach based on Gaussian processes. Asymptotic functions are used to calculate the nonadditive energy when the four helium atoms form distinct subclusters. The resulting four-body potential is used to compute the fourth virial coefficient *D*(*T*) for helium, at temperatures from 10 to 2000 K, with a path-integral approach that fully accounts for quantum effects. The results are in reasonable agreement with the limited and scattered experimental data for *D*(*T*), but our calculated results have much smaller uncertainties.

## Introduction

Standards for high-accuracy temperature and pressure metrology increasingly rely on acoustic, dielectric, or refractive measurements of gases. In recent years, the accuracy of these temperature and pressure determinations has been greatly improved by the ability to compute properties of noble gases, particularly helium, at low and moderate pressures based on ab initio quantum calculations.^1^ Example applications include a primary gas-pressure standard with relative uncertainties as small as 5 ppm (1 ppm = 10^–6^) at pressures up to 7 MPa,^2,3^ dielectric-constant gas thermometry in relation to determination of the Boltzmann constant,^4^ and refractive-index gas thermometry at temperatures below 25 K that is able to measure the thermodynamic temperature with uncertainties on the order of 0.1 mK.^5^

These first-principles methods all make use of the virial expansion, in which gas nonideality is expressed as a power series in the molar density ρ
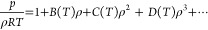
1where *p* is the pressure, *T* is the absolute temperature, and *R* is the molar gas constant. The second virial coefficient *B*(*T*) depends on the interaction between two molecules, the third virial coefficient *C*(*T*) depends on interactions among three molecules, the fourth virial coefficient *D*(*T*) depends on interactions among four molecules, and so forth.

Because the helium atom has only two electrons, modern computational chemistry techniques can compute its pair potential with extraordinary accuracy. The latest pair potential takes into account many small higher-order effects (relativistic effects, correction to Born–Oppenheimer approximation, and quantum electrodynamics) and yields interaction energies with relative uncertainties on the order of 20 ppm, with similarly small uncertainties for *B*(*T*).^6^ These uncertainties are at least 1 order of magnitude smaller than those that can be obtained from the best experiments. Calculation of *C*(*T*) requires a three-body potential. With six electrons on which to perform computations, and three dimensions instead of one, the three-body potential cannot be calculated with the same accuracy as the pair potential, but recent work^7^ has produced a surface with uncertainties on the order of 1%. The values calculated for *C*(*T*) from this three-body potential and the state-of-the-art two-body potential similarly have uncertainties more than an order of magnitude smaller than those from experiment.^7,8^

At higher pressures, the fourth virial coefficient *D*(*T*) begins to become significant. Garberoglio and Harvey^9^ calculated *D*(*T*) based on the best pair and three-body potentials available at the time, but they had to assume the four-body nonadditive contribution to be zero due to the lack of a four-body potential. A rough estimate of the uncertainty due to omission of the four-body potential was made by performing some calculations with the four-body dispersion model reported by Bade,^10,11^ which is correct in the limit of large separations. Garberoglio and Harvey observed that, due to the small magnitude of the four-body contribution, a four-body potential of only modest uncertainty (say, 20%) would be adequate for providing rigorous and relatively small uncertainties for *D*(*T*).^9^

Computing the nonadditive potential for four helium atoms, with eight electrons, is not too difficult. The more difficult part is the fitting of the potential-energy surface, which has six dimensions and must also be constrained to meet proper limits for large separations, including cases in which two or three atoms are near each other and one or two atoms are distant. For these geometries with well-separated fragments, a multipole expansion is used, where the nonadditive potential is expanded as a series in inverse powers of the large separations,^12^ using properties of the separate fragments such as charge, dipole, quadrupole, and polarizability.

However, when the four atoms are not well separated, the multipole expansion diverges and is no longer useful. Instead, a representation of the nonadditive energy is obtained as a function of the atomic positions by fitting to ab initio data. A fitting procedure requires sufficient data combined with a suitable parametric function of the six dimensions. In this work, an extensive data set is calculated from first principles, and since there are no “off-the-shelf” or intuitively reasonable functions that cover the required six-dimensional space, a machine learning method is used to interpolate the calculations.

We next present the methods that are used to calculate and fit the nonadditive potential energy of four helium atoms and the multipole expansions that are used for well-separated geometries. This is followed by a description of the path-integral calculation of the fourth virial coefficient and its uncertainty. We fit the fourth virial coefficient over a range of temperatures and compare the results to experimental data.

## Computational Methods

All electronic energies, multipoles, and polarizabilities are calculated using Molpro,^13,14^ with selected results being checked for consistency using version 2.1 of CFOUR^15^ and a “Quantum” program written at the University of Nottingham. Energies are reported in hartree, *E*_h_ ≈ 4.3597 × 10^–18^ J, and distances in bohr, *a*_0_ ≈ 5.29177 × 10^–11^ m. The four-body nonadditive energy of four helium atoms (He_4_) is defined as
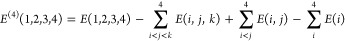
2where *E* are electronic energies calculated using the full He_4_ basis set so that a counterpoise correction is applied. The notation *E*^(4)^(1,2,3,4) is abbreviated as *E*^(4)^ for convenience.

For each required position of the four atoms of He_4_, the geometry is placed into a category according to the six internuclear distances *r*_*ij*_, and geometries in different categories are treated differently.

If at least one *r*_*ij*_ is less than *r*_short_ = 3 *a*_0_, then *E*^(4)^ is set to zero. The Boltzmann weighting of these geometries is sufficiently small that neglecting *E*^(4)^ has no significant effect on the thermodynamic quantities presented here.

Otherwise, the geometry is classified based on the distribution of “close” pairs (*i*, *j*), with *r*_*ij*_ ≤ *r*_long_. If all atoms are mutually connected by chains of close pairs, then *E*^(4)^ is calculated by interpolation (details below). The distance *r*_long_ = 7 *a*_0_ is chosen using two criteria. First, *E*^(4)^ is small (often of order 10^–10^*E*_h_ or less) when two subclusters are separated by *r*_long_. Second, the electron exchange part of *E*^(4)^ in such geometries is usually a small fraction of the total *E*^(4)^, which indicates that the overlap between the electrons of the subclusters can be neglected and that an asymptotic function is suitable for evaluating the energy (details below). This electron exchange energy is defined as the difference between *E*^(4)^ and the Coulomb part of *E*^(4)^, and the Coulomb energy is calculated using the in-house Nottingham “Quantum” program by treating electrons in different subclusters as distinguishable.

For “connected” He_4_ clusters, *E*^(4)^ is precalculated at a set of training geometries using standard quantum chemistry methods and interpolated to the required geometry. Electron correlation is modeled using coupled-cluster theory with single, double, and perturbative triple excitations, CCSD(T). The CCSDT and CCSDT(Q) methods were compared with CCSD(T) for a few geometries, but they greatly increase the computation time and do not change the energy significantly compared to the uncertainties discussed later. Training geometries are obtained using extensive “low-level” calculations with the aug-cc-pVTZ basis set, and calculations using the aug-cc-pVQZ basis set at those geometries then yield the final “high-level” interpolated *E*^(4)^.

The magnitude of *E*^(4)^ varies widely over the set of connected clusters, with a maximum magnitude of approximately 1.6 × 10^–3^*E*_h_ for a regular tetrahedron with a side length *r*_short_. The interpolation must be suitable for compact clusters like this and for extended clusters with pair distances approaching *r*_long_, where *E*^(4)^ is often around 7 orders of magnitude smaller, but the volume of physically accessible configuration space is much larger. The extent of a cluster is represented by a quantity *P*_6_, defined as *P*_6_ = ∏_*i*<*j*_(*r*_*ij*_/*r*_short_), where 1 ≤ *P*_6_ < 1936.61 for connected clusters. The following ranges of *P*_6_ are considered separately: 1 to 2 (region 1), 2 to 4 (region 2), 4 to 8 (region 3), 8 to 16 (region 4), 16 to 32 (region 5), 32 to 64 (region 6), 64 to 128 (region 7), 128 to 256 (region 8), and >256 (region 9). Regions 4 to 9 are further divided into four subregions (A, B, C, and D), giving a total of 27 subregions (1, 2, 3, 4A, 4B, etc.), and interpolation within each subregion is based on a separate data set. The subregions (A, B, C, and D) are defined as follows (after numbering the four He atoms in a permutation-invariant way, to ensure that the final energy function respects the 24-fold permutation symmetry). Subregion A: the three shortest pair distances are *r*_12_ < *r*_13_ < *r*_14_. Subregion B: the three shortest pair distances are *r*_12_ < *r*_13_ < *r*_34_ or *r*_13_ < *r*_12_ < *r*_34_. Subregion C: the three shortest pair distances are *r*_12_, *r*_13_, and *r*_23_. Subregion D: the two shortest pair distances are *r*_12_ < *r*_34_. This procedure for division into subregions is chosen from several possibilities as the one giving the best compromise between interpolation accuracy and computer time.

The interpolation method closely follows previously reported work^16^ on nonadditive interactions. A reference set and test set, each containing 5000 data points, are chosen in each subregion. Active learning is used to choose a subset of the reference set as the training set, the resulting training set is interpolated, and the interpolating function is compared with the test set. Interpolation is performed by Gaussian process (GP) regression.^17^ The active learning process starts with a single point (the energy of the highest magnitude) in the training set, then selects the worst-predicted point in the reference set and adds it to the training set at each step.

In the current work, the GP uses a zero mean function, and the kernel is a simple (not symmetrized) product of one-dimensional squared exponential kernels in each coordinate, each with a different length scale. These length scales and the noise variance constitute the hyperparameters of the GP and are chosen by maximizing the marginal likelihood of the model.^17^ The noise variance (nugget) is constrained to be no more than 10^–24^*a*_0_^2^ to prevent active learning from selecting very close data points.

Reference sets and test sets are based on randomly selected points. To improve the fitting for geometries close to the global minimum, a few regular tetrahedral geometries are added to the reference set. In regions 6 to 9, it is found that choosing reference and test points based on inverse interatomic distances does not adequately sample phase space, and unbiased sampling is used instead, with each point in 12-dimensional Cartesian space being equally probable. In each subregion, six coordinates *x*_1_ to *x*_6_ defined as *r*_ij_^–3^ are used for the regression; this is found to work better than the more conventional choice of *r*_ij_^–1^. A seventh coordinate, *x*_7_ = *P*_6_^–1/2^, is added. In the B subregions, an eighth coordinate  is also used to aid the interpolation. The D subregions tend to have *E*^(4)^ values larger than the other subregions, and for larger *P*_6_ this is attributed in part to the atoms forming two He_2_ moieties, which can interact via a quadrupole–quadrupole interaction. An eighth coordinate related to this interaction is used in the D subregions
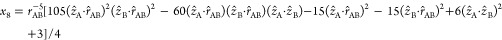
3where  is a unit vector pointing from nucleus 1 to 2 (3 to 4), and a vector from the geometric center of 1–2 to the geometric center of 3–4 has length *r*_AB_ and direction .

Details of the data sets for the subregions are given in Table 1. The active learning is terminated in each subregion once the number of training points (shown in the table) is sufficient to ensure that the interpolation error is not significantly greater than the difference between the two basis sets. This is achieved by using fewer than 10% of the reference set as training data. The more compact subregions 1 to 3 are easier to fit to a given percentage accuracy, whereas the more extended subregions are more difficult and require more training points. This may be because they cover a larger amount of configuration space, or because *E*^(4)^ fluctuates more between negative and positive values, or because the rms values may be approaching the numerical precision of the quantum chemical calculations.

**Table 1:** Details of the Subregions Used for Interpolation^a^

subregion	points	*E*^(4)^_rms_/*E*_h_	σ_fit_/*E*_h_	σ_TZ_/*E*_h_
1	125	6.6[−4]	9.9[−7]	5.3[−6]
2	230	2.8[−4]	7.1[−7]	2.1[−6]
3	275	7.4[−5]	6.4[−7]	6.7[−7]
4A	205	6.9[−6]	1.1[−7]	1.1[−7]
4B	367	1.4[−5]	6.4[−8]	1.9[−7]
4C	257	6.8[−6]	7.0[−8]	1.1[−7]
4D	332	1.7[−5]	1.1[−7]	2.2[−7]
5A	281	7.3[−7]	1.3[−8]	2.0[−8]
5B	391	1.9[−6]	2.2[−8]	4.5[−8]
5C	330	7.7[−7]	1.5[−8]	2.3[−8]
5D	478	2.8[−6]	3.2[−8]	6.4[−8]
6A	392	5.7[−8]	1.0[−9]	2.6[−9]
6B	408	2.2[−7]	3.8[−9]	9.1[−9]
6C	497	6.6[−8]	1.4[−9]	3.9[−9]
6D	382	4.9[−7]	1.7[−8]	1.5[−8]
7A	373	3.4[−9]	1.3[−10]	4.0[−10]
7B	491	3.1[−8]	6.2[−10]	2.1[−9]
7C	441	4.5[−9]	2.5[−10]	7.3[−10]
7D	495	8.7[−8]	2.9[−9]	3.2[−9]
8A	304	4.7[−10]	2.6[−11]	5.0[−11]
8B	410	6.5[−10]	3.6[−11]	1.8[−10]
8C	409	7.9[−10]	3.9[−11]	1.6[−10]
8D	384	5.2[−9]	1.2[−11]	4.3[−10]
9A	97	1.8[−10]	1.3[−11]	1.8[−11]
9B	363	3.6[−10]	2.0[−11]	1.0[−10]
9C	87	4.9[−10]	1.1[−11]	5.2[−11]
9D	199	4.2[−10]	9.0[−12]	5.3[−11]

a *E*^(4)^_rms_ is the rms of the calculated *E*^(4)^ values, σ_fit_ is the rms error over an independent test set, and σ_TZ_ is the rms difference between “low-level” and “high-level” energies. Square brackets denote powers of 10; for example, 6.6[−4] means 6.6 × 10^–4^.

Overall, *E*^(4)^ decreases with increasing cluster extent *P*_6_, as expected. For compact clusters, such as regular tetrahedra with short bond lengths, the energy is positive. For more extended geometries (including regular tetrahedra near the global He_4_ energy minimum), it has positive and negative values; the positive values tend to be larger and cover more configuration space. The most negative *E*^(4)^ values are associated with planar Y-shaped geometries. The difference between aug-cc-pVTZ and aug-cc-pVQZ calculations increases with increasing *P*_6_, relative to the magnitude of the energy.

The uncertainty in fitted *E*^(4)^ consists of the fitting error and the approximations inherent in the quantum chemistry calculations. The latter cannot be calculated exactly, instead, an uncertainty is associated with each subregion by considering the quantities σ_fit_ and σ_TZ_, given in Table 1, to be independent errors. The use of σ_TZ_ as a (conservative) estimate of the uncertainty in the calculated energy is supported by performing a few higher-level calculations at selected geometries and by comparison with analogous calculations on He_3_.^18^ The combined uncertainty  is then expressed as a percentage of *E*_rms_^(4)^, and the resulting value is used as the percentage uncertainty in that subregion. These uncertainty estimates are checked by comparing the final fitted energy (which is calculated at the aug-cc-pVQZ level) with aug-cc-pVTZ calculations over each test set. The resulting rms error is found to be very similar to the uncertainty estimate in every subregion, which also indicates that the transfer learning (using aug-cc-pVTZ training points for interpolating aug-cc-pVQZ calculations) does not introduce significant additional errors.

Since *E*^(4)^ is fitted separately in each subregion, the fitted function is not continuous across region boundaries, and the discontinuity may be substantial since the fitting error is likely to be largest at the boundaries. These discontinuities do not affect the calculation of virial coefficients; therefore, no attempt is made to remove them. However, it would not be advisable to perform calculations that relied on forces calculated from the fitted energy. Fitting errors tend to be equally divided between over- and underestimates of the calculated energy, which means that substantial cancellation of the fitting errors is expected in the calculation of the virial coefficients.

For He_4_ clusters that are not “connected”, asymptotic functions are used to approximate the nonadditive energy. Brief details are given next. Since *E*^(4)^ in these regions is very small, decreases rapidly with cluster size, and has substantial cancellation between positive and negative regions, the asymptotic functions do not need to be highly accurate and they are calculated using only charges, dipoles, and dipole excitations on each atom. The resulting uncertainty in *E*^(4)^ is estimated to be 10%, based on comparison with calculations at selected geometries near the boundary with the “connected” region. The main source of error arises from neglecting quadrupoles and higher multipoles, although there are also some approximations in the calculation of the dipole properties. The percentage error is generally expected to decrease with an increasing cluster size.

When all six interatomic distances are greater than *r*_long_, eq 2 is used to define *E*^(4)^. Each atom is represented using a set of ten pseudostates:^19^ the ground state and nine excited states corresponding to three excitations in each of the *x*, *y*, and *z* directions. The excited states are given fixed excitation energies of 0.818, 1.048, and 2.296 *E*_h_, which are chosen from a fit to a large set of cluster polarizabilities. The dipole oscillator strengths for each pseudostate are obtained from a fit to the imaginary-frequency-dependent dipole polarizability α(iω), calculated using time-dependent CCSD theory with the aug-cc-pVQZ basis set at 11 ω values. The energies in eq 2 (relative to the energy of noninteracting atoms) are then calculated as the lowest eigenvalue of a sparse Hamiltonian matrix , where *p* and *q* are pseudostates of each atom 1 to 4, and the matrix elements include diagonal excitation energies and off-diagonal point dipole–dipole interactions.

When one interatomic distance (*r*_12_) is below *r*_long_, an asymptotic function is calculated based on the three well-separated moieties 1–2, 3, and 4. The nonadditive energy is written as
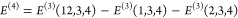
4where *E*^(3)^(*a*, *b*, *c*) is the three-body nonadditive energy *E*(*a*, *b*, *c*) – *E*(*a*, *b*) – *E*(*a*, *c*) – *E*(*b*, *c*) + *E*(*a*) + *E*(*b*) + *E*(*c*). The nonadditive induced dipole interactions are modeled as described above, and the polarizabilities of atoms 1 and 2 are each taken to be half of the polarizability of the 1–2 moiety, which is fitted as a function of bond length *r*_12_. The 1–2 moiety also has a quadrupole, which is calculated by using CCSD theory with the aug-cc-pVQZ basis set and fitted as a function of *r*_12_. It differs by less than 0.001 e *a*_0_^2^ from accurate literature calculations for all bond lengths.^20^ The fitted quadrupole θ is then represented as opposing dipoles μ = θ/(2*r*_12_) on atoms 1 and 2. The Hamiltonian matrix includes interactions of these permanent atomic dipoles with the pseudostates of atoms 3 and 4. Atoms 1 and 2 are assumed not to polarize each other (although for other atoms and molecules where polarization is more important, it would be advisable to include some mutual polarization in the asymptotic model).

When two interatomic distances involving the same atom (*r*_12_ and *r*_13_) are below *r*_long_, regardless of the distance *r*_23_, an asymptotic function is calculated based on the two moieties 1–2–3 and 4. The nonadditive energy is written as
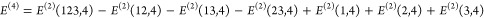
5where *E*^(2)^(*a*, *b*) is the two-body nonadditive energy *E*(*a*, *b*) – *E*(*a*) – *E*(*b*). The nonadditive induced dipole interactions are modeled as described above, and the nonadditive contributions to the polarizabilities of atoms 1 to 3 within the 1–2–3 moiety are each taken to be one-third of the total nonadditive polarizability of 1–2–3, which is fitted as a function of the three bond lengths. The 1–2–3 moiety also has a dipole and quadrupole, which are calculated by using CCSD theory with the aug-cc-pVQZ basis set. The fitted dipole, which is entirely nonadditive, is represented uniquely as a charge on each atom. The quadrupoles of the isolated pairs 1–2, 1–3, and 2–3 are calculated as described above, and the remaining nonadditive quadrupole of 1–2–3 is then represented as three additional pairs of opposing dipoles on each pair of atoms 1–2, 1–3, and 2–3; this is also a unique definition, except for geometries when the atoms are exactly collinear, which are not used in the data set. The resulting atomic charges and dipoles are fitted as a function of the bond lengths. A counterpoise correction is not used for calculating nonadditive multipoles or nonadditive polarizabilities. The Hamiltonian matrix includes interactions of these permanent atomic charges and dipoles with the pseudostates of atom 4. Atoms 1, 2, and 3 are assumed not to polarize each other. This asymptotic function involves a dipole–induced dipole interaction energy, which is always negative and decreases as the inverse sixth power of the distance from 1–2–3 to 4. This could be an important long-ranged contribution to the fourth virial coefficient since the energy falls off relatively slowly with distance, but in practice, the dipole of He_3_ is small, with a maximum of only ≈0.002 e *a*_0_ for the most compact right-angled trimers with two bond lengths near *r*_short_.

Finally, when two interatomic distances *r*_12_ and *r*_34_ are below *r*_long_, an asymptotic function is calculated based on the two moieties 1–2 and 3–4. The nonadditive energy is written as
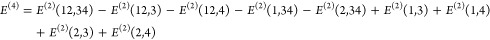
6The polarizabilities and quadrupoles of 1–2 and 3–4 are modeled as described above. The Hamiltonian matrix includes interactions of the permanent atomic dipoles and pseudostates of atoms 1 and 2 with those of atoms 3 and 4. Atoms in a pair, (1,2) and (3,4), are assumed not to polarize each other. This asymptotic function involves a quadrupole–quadrupole interaction energy, which decreases as the inverse fifth power of the distance from 1–2 to 3–4. This is the largest contribution to the energy at long range (see also the discussion of D subregions above), but its contribution to the fourth virial coefficient is not expected to be large because regions of positive and negative nonadditive energy will cancel each other.

### Path-Integral Calculation of *D*(*T*)

The calculation of the fourth virial coefficient *D*(*T*) followed the procedure outlined in refs (1) and (9) using the path-integral formulation of quantum statistical mechanics. In this approach, each quantum particle is represented by a ring polymer of *P* monomers (beads). The virial coefficient is written as
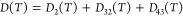
7where *D*_2_(*T*) is the value obtained considering the pair potential only, *D*_32_(*T*) is the difference between *D*(*T*) computed with the three- and two-body potential and *D*_2_(*T*); expressions for these quantities can be found in ref (9). Analogously, *D*_43_(*T*) is the contribution to *D*(*T*) from the nonadditive four-body interaction, and it is given by the infinite volume (*V*) limit of
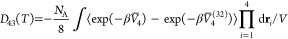
8where *N*_A_ is the Avogadro constant,*V*_4_ is the total four-body interaction potential, and *V*_4_^(32)^ is the interaction potential of four atoms excluding the nonadditive four-body contribution. In eq 8, the average ⟨·⟩ is performed over the configuration of ring polymers sampled according to the path-integral prescription, and **r**_*i*_ is the position of the first bead of the ring polymer associated with particle *i*. The overbar represents the average interaction potential among the ring polymers, as specified by the path-integral approach.^1^ We performed the integration over the coordinates **r**_*i*_ using the VEGAS Monte Carlo algorithm as implemented in the Cuba library,^21^ using 10^6^ evaluations. The average ⟨·⟩ over the ring polymers was evaluated by drawing eight independent configurations at each sampling point. We used the same value of *P* as in ref (9), that is, *P* = nint(4 + 620/(*T*/1 K)^0.7^), where nint(*x*) denotes the nearest integer to *x*. We checked that we obtain the same results increasing *P* by 30% at 10, 120, and 1000 K. We performed as many independent runs as needed so that the statistical uncertainty of *D*_43_(*T*) and *D*_32_(*T*) (evaluated as the variance of the mean) became smaller than the propagated uncertainty coming from the four-body and three-body potential, respectively.

The propagation of the uncertainty of the potentials to the uncertainty in *D*(*T*) was performed using the functional-differentiation approach.^1^ In particular, we have
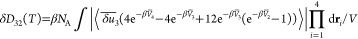
9
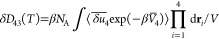
10where δ*u*_*n*_ is the estimated standard (*k* = 1) uncertainty of the nonadditive *n*-body potential, and, as above, ⟨·⟩ indicates an average of ring polymers. Integration of eqs 9 and 10 was performed analogously to the integrations *D*_43_ and *D*_32_. In this case, however, we found properly converging results using only one run with 5 × 10^5^ Monte Carlo evaluations.

Table 2 reports the values of the uncertainty of *D*(*T*) propagated from the uncertainties of the potentials. With respect to previous calculations, the use of an improved three-body potential^7^ resulted in a reduction of the corresponding propagated uncertainty by a factor of approximately 4 across the whole temperature range investigated here. Nevertheless, the largest contribution to the uncertainty of *D*(*T*) comes from the propagated uncertainty from the four-body potential. In a previous work, this unknown contribution was estimated on the basis of a simple model for the four-body interaction. Actual uncertainties are a bit smaller than those expected at temperatures *T* ≳ 80 K but larger than the previous estimate by up to a factor of 2 at temperatures down to *T* = 10 K. This revised estimate of the uncertainty is likely to be an overestimate because there is expected to be significant cancellation of errors between regions where the fit is too high and regions where it is too low. We also note that the integral in eq 8 has positive and negative contributions that are each about 10 times larger in magnitude than the total.

**Table 2:** Contributions to the Standard (*k* = 1) Uncertainty of *D*(*T*) Propagated from the Potentials^a^

temperature (K)	*u*(*V*_2_) (cm^9^/mol^3^)	*u*(*V*_3_) (cm^9^/mol^3^)	*u*(*V*_4_) (cm^9^/mol^3^)	*u*_pot_(*D*) (cm^9^/mol^3^)
10	16.90(6)	19.3(2)	91(2)	94
15	4.83(1)	7.52(6)	53.0(7)	54
20	2.088(5)	4.04(2)	35.1(5)	35
24.5561	1.232(4)	2.72(2)	29.0(4)	29
30	0.707(3)	1.93(2)	22.7(3)	23
50	0.1843(7)	0.819(5)	13.2(2)	13
80	0.0622(3)	0.444(4)	7.8(1)	8
120	0.0271(1)	0.286(2)	5.54(9)	6
173.15	0.01352(6)	0.201(1)	4.07(9)	4
200	0.01045(5)	0.179(1)	3.63(5)	4
223.15	0.00868(4)	0.165(1)	3.22(6)	3
250	0.00716(3)	0.151(1)	2.97(7)	3
273.16	0.00622(3)	0.141(1)	2.77(4)	3
300	0.00537(3)	0.134(9)	2.48(6)	2
323.15	0.00476(2)	0.126(8)	2.30(3)	2
400	0.00343(1)	0.112(1)	1.96(2)	2
500	0.002469(8)	0.0981(8)	1.61(4)	1.6
700	0.001525(5)	0.0806(7)	1.21(1)	1.2
1000	0.000942(3)	0.0675(5)	0.91(1)	0.9
1500	0.000548(2)	0.0543(5)	0.67(1)	0.7
2000	0.000375(2)	0.0425(4)	0.551(8)	0.6

a *u*(*V*_2_): pair potential,^6^*u*(*V*_3_): three-body potential,^7^*u*(*V*_4_): four-body potential of this work. The numbers in parentheses are the standard uncertainties from the PIMC calculation. The last column reports the total standard (*k* = 1) uncertainty, obtained as the sum in quadrature of the three contributions.

Table 3 reports our calculated values for *D*(*T*), including all the contributions from the various nonadditive potentials (see eq 7). The contribution *D*_2_(*T*), obtained considering only pairwise additive interactions, uses the same pair potential as ref (9) and has not been recalculated. However, we recomputed the contribution of *D*_32_(*T*) due to the three-body potential. As already noted in the case of the third virial coefficient,^7^ the updated three-body potential results in a systematic negative shift in *D*_32_(*T*). As expected, however, the updated three-body contribution to *D*(*T*) is compatible with that in ref (9) within mutual uncertainties.

**Table 3:** Values of the Various Contributions to *D*(*T*) from Equation 7^a^

temperature (K)	*D*_2_(*T*) (cm^9^/mol^3^)	*D*_32_(*T*) (cm^9^/mol^3^)	*D*_43_(*T*) (cm^9^/mol^3^)	*D*(*T*) (cm^9^/mol^3^)
10	4515(50)	–363(6)	17(3)	4169 ± 214
15	3056(20)	–165(2)	11(1)	2902 ± 114
20	2854(20)	–86.4(9)	8.9(8)	2777 ± 78
24.5561	2780(10)	–56.4(6)	7.0(7)	2730 ± 64
30	2701(6)	–36.7(4)	5.4(5)	2669 ± 47
50	2275(2)	–19.3(2)	2.5(3)	2258 ± 27
80	1799.0(7)	–18.6(1)	1.1(2)	1781 ± 16
120	1402.3(5)	–21.53(7)	0.5(9)	1381 ± 11
173.15	1089.4(4)	–23.82(5)	0.4(1)	1066 ± 8
200	979.4(3)	–24.65(5)	0.2(6)	955 ± 7
223.15	901.2(3)	–25.19(5)	0.2(7)	876 ± 6
250	825.2(3)	–25.59(4)	0.3(5)	800 ± 6
273.16	768.9(3)	–25.85(4)	0.2(5)	743 ± 6
300	712.9(3)	–26.16(4)	0.4(5)	687 ± 5
323.15	671.0(2)	–26.31(4)	0.4(5)	645 ± 5
400	560.2(2)	–26.60(3)	0.4(4)	534 ± 4
500	460.3(2)	–26.66(3)	0.6(3)	434 ± 3
700	336.9(2)	–26.24(3)	0.8(2)	311 ± 2
1000	236.6(1)	–25.21(2)	1.0(2)	212 ± 2
1500	153.75(9)	–23.45(2)	1.2(2)	131.6 ± 1.4
2000	110.83(7)	–21.95(2)	1.3(1)	90.3 ± 1.1

a Numbers in parentheses are standard (*k* = 1) statistical uncertainties from the PIMC calculation. The last column reports our best values for *D*(*T*), with expanded (*k* = 2) uncertainties that include the uncertainty propagated from the potentials (see Table 2).

The computed values of the four-body contribution to *D*(*T*), *D*_43_(*T*) of eq 7, are found to be positive. They decrease from *T* = 2000 K down to *T* = 273.16 K and increase again at lower temperatures. We notice that the *D*_43_ values are smaller than the propagated uncertainty from the four-body potential except at the highest temperatures. This is due to the fact that *D*_43_(*T*) is obtained by integrating a function with positive and negative regions (see eq 8), while *u*(*V*_4_) is obtained by integrating a strictly positive function, as seen in eq 10, together with the fact that the uncertainty δ*u*_4_ is a sizable fraction of the absolute value of the four-body potential.

Our values of *D*(*T*) as shown in Table 3 are fully consistent within mutual uncertainties with those given in ref (9) and have similar uncertainties. The main advance in the present work (in addition to the use of an improved three-body potential) is the rigorous inclusion of the nonadditive four-body interaction and its uncertainty, allowing us to produce values with no contributions ignored and with a complete uncertainty budget.

### Correlation for *D*(*T*)

We developed a correlation for the values of *D*(*T*) reported in Table 3 of the form
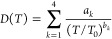
11using *T*_0_ = 100 K. The values of the coefficients *a*_*k*_ and *b*_*k*_ are reported in Table 4. The function in eq 11 passes within the expanded statistical uncertainties in *D*, *U*_stat_(*D*), in the temperature range 10–2000 K, with the exception of 15 K, where it deviates from the calculated value of *D*(*T*) by 1.06 expanded statistical uncertainties. This function extends in a reasonable way down to the temperature where *D*(*T*) attains its maximum (*T* ∼ 5 K), but at this point, the deviation from the simulation data reported previously^9^ increases to 2 expanded statistical uncertainties.

**Table 4:** Parameters for Equation 11^a^

*k*	*a*_*k*_ (cm^9^/mol^3^)	*b*_*k*_
1	204.153	2.07032
2	–1067.96	1.55831
3	2678.59	0.857992
4	–257.562	0.296535

a The *a*_*k*_ have dimensions of (cm^9^/mol^3^), while *b*_*k*_ are dimensionless. The quantity *T*_0_ was set to 100 K.

### Comparison with Experimental Data

Experimental measurement of *D*(*T*) requires high-accuracy density measurements up to high pressures, and the reported experimental values for helium have relatively high uncertainties and, in some cases, are mutually inconsistent.

**Figure 1:**
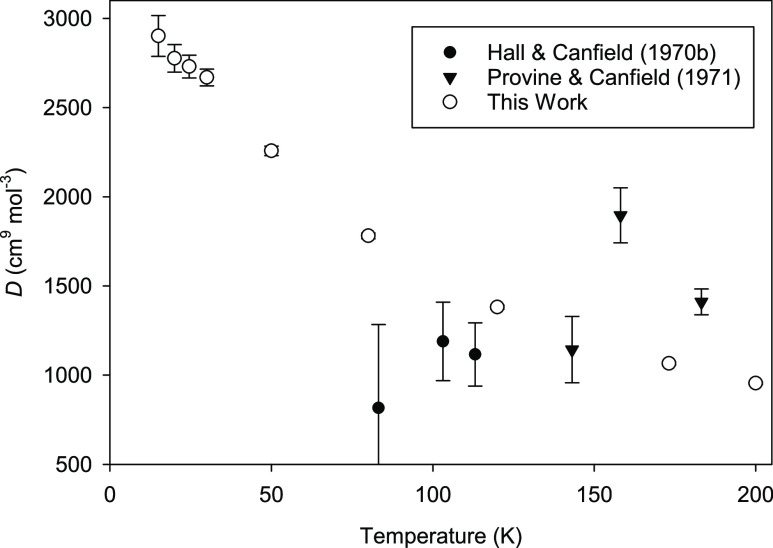
Comparison of calculated *D*(*T*) for ^4^He at low temperatures with experimental results.^22,23^ Error bars represent expanded uncertainties with a coverage factor *k* = 2. Expanded uncertainties for this work are smaller than the size of the symbols at and above 50 K.

Figure 1 displays our results at temperatures of 200 K and below. No experimental *D*(*T*) exist below 83 K, and the two experimental sources^22,23^ show some scatter and an unclear trend with temperature. Our results have much smaller expanded uncertainties than the experiments (smaller than the size of the symbols above 50 K). They are consistent in magnitude with the reported experimental values but show a clear temperature trend that could not be discerned by inspection of the experimental points.

**Figure 2:**
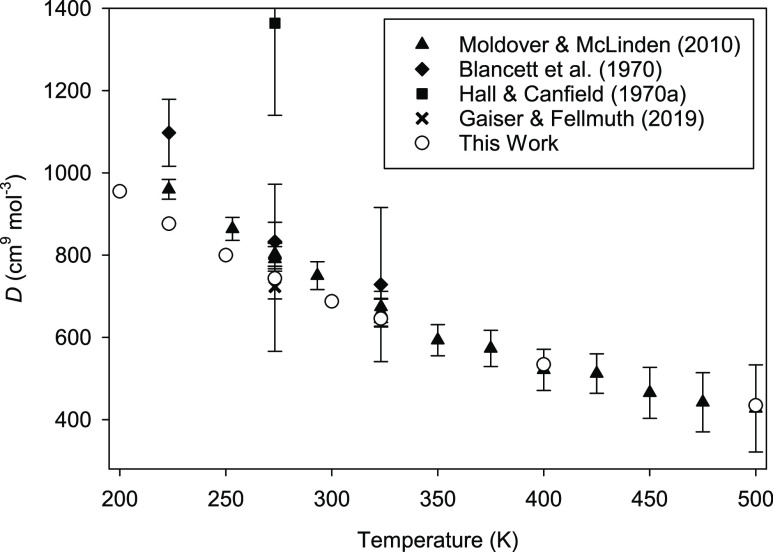
Comparison of calculated *D*(*T*) for ^4^He at high temperatures with experimental results.^24−27^ Error bars represent expanded uncertainties with coverage factor *k* = 2. Expanded uncertainties for this work are smaller than the size of the symbols.

The experimental situation is better at higher temperatures, due to the recent results reported by Moldover and McLinden^24^ and by Gaiser and Fellmuth.^25^ These data, along with those from two older studies,^26,27^ are plotted along with our results in Figure 2. The point derived by Gaiser and Fellmuth^25^ from dielectric-constant gas thermometry at 273.16 K has relatively large error bars but is in good agreement with our results. The agreement with values reported by Moldover and McLinden^24^ above about 275 K is excellent, but there is a systematic offset at lower temperatures. This offset is not large, but it is outside the mutual expanded uncertainties. In ref (9), it was speculated that this might be due to an unrecognized error (such as a small error in calibration of the sinker used) in the experiments described in ref (24). However, a recent analysis^1^ suggests that the discrepancy instead arose from the use of a truncated virial expansion to obtain the fourth virial coefficient in ref (24) when the contribution of the fifth and sixth virial coefficients, while small, was not entirely negligible.

## Conclusions

We have used GPs within an active learning approach to interpolate accurate ab initio values for the nonadditive four-body potential of a set of four helium atoms. The resulting surface is supplemented by long-range functions that exhibit proper asymptotic behavior, including cases where two or three molecules are clustered together with the other(s) at a large distance. To the best of our knowledge, this is the first complete four-body potential ever presented for helium.

The four-body surface allows us to perform the first calculation of the fourth virial coefficient of helium with a complete uncertainty budget, which is necessary for the use of helium in gas metrology. This calculation also employs the state-of-the-art two-body and three-body potentials; the use of the latest three-body potential^7^ reduces the uncertainty due to that source compared to the calculations of ref (9). The resulting values for *D*(*T*) have significantly lower uncertainties than any values derived from experiment.

Because of the very high accuracy of the state-of-the-art two- and three-body potentials used, the greatest source of uncertainty in the *D*(*T*) values presented here comes from the four-body potential. Reduction of this contribution to the uncertainty would require the use of larger basis sets (such as aug-cc-pV5Z), a higher level of electron correlation (such as CCSDT(Q)), a more accurate interpolation which would most likely require more data points, and possibly consideration of relativistic effects. This is beyond our current computational capabilities.

Because the new four-body potential requires substantially more computing time than, for example, the three-body potential, our calculations could only be performed down to 10 K. With more computational resources, they could be extended to lower temperatures, although below about 7 K it would also be necessary to include exchange effects as derived in ref (9). However, because the four-body contribution to *D*(*T*) is relatively small (see Table 3), the results from ref (9) that assumed *D*_43_ = 0 and performed calculations down to 2.6 K should be a reasonable approximation for temperatures below 10 K.

## Supporting Information Available

Description of the other files in the Supporting Information (TXT)
See README.txt for description (TXT)
See README.txt for description (TXT)
Fortran code, data files and build script. See README.txt for description (ZIP)
